# Protective effect of paraoxonase 1 gene variant L55M in retinal vein occlusion

**Published:** 2013-02-25

**Authors:** Huseyin Ortak, Erkan Söğüt, Ömer Ateş, Ünal Erkorkmaz, İsmail Benli, Ali Akbas, Selim Demir, Hüseyin Özyurt

**Affiliations:** 1 Gaziosmanpasa University Faculty of Medicine, Department of Ophthalmology, Tokat, Turkey; 2 Gaziosmanpasa University Faculty of Medicine, Department of Biochemistry, Tokat, Turkey; 3 Gaziosmanpasa University Faculty of Medicine, Department of Medical Biology, Tokat, Turkey; 4 Gaziosmanpasa University Faculty of Medicine, Department of Biostatistics, Tokat, Turkey

## Abstract

**Purpose:**

To determine if the paraoxonase 1 L55M and paraoxonase 1 Q192R gene polymorphisms have an effect on the risk of having a retinal vein occlusion (RVO).

**Methods:**

This case-control prospective study included 120 patients with RVO and 84 control subjects. All subjects were screened for age, gender, hypertension, diabetes, body mass index, fibrinogen, high-density lipoprotein cholesterol, low-density lipoprotein cholesterol, triglycerides, total cholesterol, and very low-density lipoprotein. Subjects were also questioned about their smoking habits. Genomic DNA was extracted from peripheral leukocytes from EDTA anticoagulated blood. Genotyping of the paraoxonase 1 L55M and paraoxonase 1 Q192R polymorphisms was performed using real-time PCR.

**Results:**

The frequency of the paraoxonase 1 (*PON1*) 55 LL genotype was significantly lower in patients with RVO than in the control subjects (28% versus 55%; p=0.005). Logistic regression analyses were also conducted. After adjusting for gender, diabetes, hypertension, plasma fibrinogen levels, and high-density lipoprotein cholesterol, the lower LL genotype was found to be an independent predictor of RVO (β=1.755; odds ratio=5.783; p<0.001; 95% confidence interval=2.579–12.967).

**Conclusions:**

Subjects with a lower frequency *PON1* 55 LL genotype had a higher risk of RVO. These results indicate that paraoxonase gene polymorphisms may be a possible risk factor for RVO. We suggest that the LL genotype may have a protective role in the pathogenesis of RVO in the Turkish population.

## Introduction

Retinal vein occlusion (RVO) is one of the important retinal vascular diseases that visual acuity may complicate with retinal hemorrhage, edema, and neovascularization in the aging population. In a study of data pooled from this population, the prevalence of RVO was 5.20 cases per 1,000 for RVO in general, 4.42 cases per 1,000 for branch retinal vein occlusion (BRVO), and 0.8 cases per 1,000 for central retinal vein occlusion (CRVO) [1]. Several studies have shown that factors such as hypertension, coagulation disorders, diabetes, hyperlipidemia, genetics, smoking, and glaucoma are associated with RVO [2]. Oxidative stress may also contribute to the development of RVO [3]. A recent study reported that young patients with CRVO have low levels of serum paraoxonase-1 arylesterase activity, which is associated with increased oxidative stress [4]. *PON1* is secreted by liver cells and bound to high-density lipoproteins (HDLs) while in circulation. *PON1* has antioxidant enzyme capacity, including peroxidase-like activity and the ability to hydrolyze platelet-activating factor. Dysfunction of *PON1* can cause lipid peroxidation and vascular disease development [5]. In addition, *PON1* has been reported to affect atherogenesis by preventing low-density lipoprotein (LDL) oxidation and preserving the function of HDL [6]. The *PON1* gene has two exonic polymorphisms in the coding region at positions 55(L/M) and 192(Q/R); these polymorphisms have been defined as the molecular basis for interindividual variability [7]. An association between *PON1* polymorphisms and cardiovascular disease has been found [8], and a variant of the *PON1* gene is associated with diabetic retinopathy in insulin-dependent diabetes mellitus [9]. In this context, RVO has many shared causative factors with diabetes and cardiovascular diseases, such as lipid disorders and oxidative stress [10]. In the present study, we aimed to determine whether the *PON1* L55M and Q192R polymorphisms are risk factors for the etiopathogenesis of RVO.

## Methods

### Study population

A total of 204 subjects who presented to the ophthalmology clinic at Gaziosmanpasa University Hospital were enrolled in the Turkish study population. The study population consisted of 120 patients diagnosed with RVO using fundus fluorescein angiography and ophthalmic examinations and 84 age-matched controls without RVO. Subjects with trauma, cancer, anticoagulation therapy, or abnormal liver or renal functional tests were excluded from this study. A full ophthalmological evaluation was performed on each subject. All subjects were screened for age, gender, hypertension (HT), diabetes (DM), and body mass index (BMI). Serum levels of fibrinogen, high-density lipoprotein cholesterol (HDL-C), low-density lipoprotein cholesterol (LDL-C), triglycerides (TG), total cholesterol (TC), and very low-density lipoprotein cholesterol (VLDL-C) were also determined. All subjects were also questioned about their smoking habits. The hospital ethics committee approved the study, and written informed consent was obtained from each patient after the nature and purpose of the study were fully explained. All experiments were performed in accordance with the Declaration of Helsinki.

### Determination of clinical characteristics

Hypertension was defined as systolic blood pressure ≥140 mmHg and diastolic blood pressure ≥90 mmHg. Diabetes mellitus was defined according to the American Diabetes Association (ADA) diagnostic criteria [11]. BMI was calculated as weight (kg) divided by height squared (m^2^).

### Biochemical analysis

Fasting blood samples were obtained via peripheral venous puncture from all subjects. Citrated plasma and serum samples were separated by centrifugation (1500 × *g* for 10 min at 4 °C). The plasma and serum samples were separated and transferred to clean test tubes and stored at −70 °C until further analysis. Plasma fibrinogen levels were determined using the Clauss method [12] and clotting reagents from Diagnostica Stago (Asnieres, France). Serum total HDL-C, VLDL-C, TG, and LDL-C levels were analyzed with a Cobas C 6000 auto analyzer (Roche Molecular Biochemicals, Mannheim, Germany).

### Genotyping

The procedure for genotyping the polymorphisms was used as previously described [13]. *PON1* Q192R and *PON1* L55M polymorphisms were determined using real-time PCR to analyze patient and control DNA. Genomic DNA was isolated from peripheral leukocytes from EDTA anticoagulated blood using the High Pure PCR Template Preparation Kit (Roche Molecular Biochemicals). Briefly, the following previously reported [13] primers were used: 5′-TAT TGT TGC TGT GGG ACC TGA G-3′ and 5′-CCT TCT GCC ACC ACT CGA AC-3′ for the *PON1* Q192R polymorphism and 5′-CCT GCA ATA ATA TGA AAC AAC CTG-3′ and 5′-CTA GAA CAC ACA GAA AAG TGA AAG AAA AC-3′ for the *PON1* L55M polymorphism. The detection of oligonucleotide sets during *PON1* Q192R and *PON1* L55M genotyping was achieved using a 3′-fluorescein-labeled anchor and either a 5′-LC Red 705-labeled (*PON1* Q192R) sensor probe or a 5′-LC Red 640-labeled (*PON1* L55M) sensor probe. The probes for *PON1* Q192R were sensor 5′-CCC CTA CTT ACA ATC CTG GGA GAT-3′ and anchor 5′-ATT TGG GTT TAG CGT GGT CGT ATG TTG-3′ and for *PON1* L55M were sensor 5′-CTC TGA AGA CAT GGA GAT ACT GCC-3′ and anchor 5′-ATG GAC TGG CTT TCA TTA GCT CTG TGA GT-3′. The primers and the fluorescent-labeled probes were synthesized by TIB MolBiol (Berlin, Germany). PCR and melting curve analyses were performed in 20-μl glass capillaries (Hoffmann-La Roche, Mannheim, Germany) [14]. During PCR, the Roche LightCycler 1.5 real-time PCR instrument (Roche Instrument Center AG, Rotkreuz, Switzerland) and Light Cycler Software 4.05 (Roche Diagnostics GmbH, Mannheim, Germany) was used to continuously monitor the current fluorescence and fluorescence history of each capillary. Analysis of the PCR products on agarose gels and sequencing confirmed the presence of the specific PCR products. The observed melting point temperatures were 63.0 °C for *PON1* 55 MM, 58 °C for *PON1* 55 LL, 63 °C for *PON1* 192 QQ, and 57 °C for *PON1* 192 RR. Both melting point temperatures were noted in the case of subjects heterozygous for *PON1* polymorphisms (*PON1* L55M*, PON1* Q192R).

### Statistical analysis

Either the χ^2^ test or Fischer’s exact test was used to compare the distribution of the *PON1* polymorphisms of patients with RVO with those of the healthy controls. Odds ratios (ORs) and 95% confidence intervals (CIs) were calculated whenever the chi-square or Fischer’s exact test was significant. Significant probability values were also corrected for multiple testing (Bonferroni correction; Pc). The χ^2^ test was used to evaluate Hardy–Weinberg equilibrium for the distribution of the genotypes of the patients and the controls. Categorical data were expressed as counts and percentages. According to the Kolmogorov–Smirnov normality test, the two independent sample Student *t* test was used to compare continuous data between two groups. Continuous data are expressed as mean±standard deviation (SD). A multivariate logistic regression model was implemented to determine LL genotype, gender, HT, DM, HDL-C, and fibrinogen level associated with vein occlusion. P values below 0.05 were considered statistically significant. Statistical analysis was performed using commercial software (PEPI 3.0; IBM SPSS Statistics 19, SPSS Inc., Somers, NY).

## Results

The study population consisted of 120 patients with RVO who presented to the ophthalmology clinic at Gaziosmanpasa University Hospital and 84 age-matched controls. The mean age was 64.63±7.69 (age range; 50–88) years for the control group and 63.56±8.20 (age range: 32–82) years for the patient group (p=0.347). The frequency of hypertension was higher in the RVO group than in the control group (76.7% versus 39.3%, p=0.001). The baseline characteristics of the patients and controls are shown in Table 1. There was no significant difference between the patients with RVO and the control group regarding serum total cholesterol, triglycerides, VLDL-C, LDL-C, DM, BMI, or smoking status. The distribution of the genotype and allele frequencies of *PON1* L55M and *PON1* Q192R in patients with RVO and the controls is shown in Table 2. The frequency of the *PON1* 55 LL genotype was significantly lower in patients with RVO than in the control subjects (28% versus 55%; p=0.005). Among the patients with RVO, 16 patients had CRVO, and 104 patients had BRVO. The two RVO groups had significantly different genotype distributions. The RR genotype occurred more frequently in patients with CRVO than in patients with BRVO (38% and 6%, respectively; p=0.0016; Table 3). *PON1* genotype distribution was in agreement with the Hardy–Weinberg expectations in the control group but not in the RVO group (p<0.05). A multivariate logistic regression analysis was also conducted using RVO as the dependent variable and hypertension, gender, diabetes, HDL-C, and fibrinogen. We found that the frequency of the *PON1* 55 LL genotype was an independent predictor of RVO. After adjusting for gender, hypertension, fibrinogen, and frequency of diabetes, the *PON1* 55 LL genotype was found to be associated with an increased risk of RVO (β=1.755; OR=5.783; p<0.001; 95% CI=2.579–12.967). The results of the multivariate logistic regression analysis are presented in Table 4**.**

**Table 1:** Baseline characteristics of patients with retinal vein occlusion and control subjects.

**Characteristics**	**Control (n=84)**	**Vein occlusion (n=120)**	**p**
Gender (Male)	35 (41.7)	72 (60.0)	**0.010**
Age (years)	64,63±7,69	63,56±8,20	0.347
BMI (kg/m^2^)	26,23±3,53	26,02±2,42	0.637
HT n (%)	33 (39.3)	92 (76.7)	**<0.001**
DM n (%)	12 (14.3)	30 (25.0)	0.054
Smoking n (%)	25 (29.8)	51 (42.5)	0.064
HDL-C (mg/dl)	47,64±14,12	43,10±9,61	**0.011**
LDL-C (mg/dl)	141,54±42,47	134,60±26,97	0.188
Total cholesterol (mg/dl)	203,85±44,58	197,28±30,67	0.244
Triglycerides (mg/dl)	182,56±71,90	169,08±67,14	0.178
VLDL-C (mg/dl)	35,08±13,82	33,78±12,26	0.488
Fibrinogen (mg/dl)	288,24±47,49	330,08±56,29	**<0.001**

Data were shown as n (%) and mean ±standard deviation.

**Table 2:** Genotypic and allelic distribution of PON1 55L/M and PON1 192Q/R polymorphisms among patients with retinal vein occlusion and control subjects.

**PON locus**	**Control** **(n=84)**	**Vein Occlusion** **(n=120)**	**p (pc)**	**OR (95% CI of OR)**
**PON55L/M**				
L/L	46(55%)	34(28%)	**0.005 (0.015)**	
L/M	28(33%)	70(59%)		
M/M	10(12%)	16(13%)		
*Allele frequency*				
L	120(71%)	138(58%)	**0.002 (0.004)**	1.85 (0.35–0.82)
M	48(29%)	102(42%)		

**PON192Q/R**				
Q/Q	10(12%)	16(13%)	**0.03** (0.06)	
Q/R	54(64%)	92(77%)		
R/R	20(24%)	12(10%)		
*Allele frequency*				
Q	74(44%)	124(52%)	0.06	1.36 (0.91–2.02)
R	94(56%)	116(48%)		

(Bonferroni correction; Pc)

**Table 3:** Genotypic and allelic distribution of PON1 55L/M and PON1 192Q/R polymorphisms among patients with central retinal vein occlusion and branch retinal vein occlusion.

**PON locus**	**Central Retinal Vein Occlusion (n=16)**	**Branch Retinal Vein Occlusion (n=104)**	**p (pc)**	**OR (95% CI of OR)**
**PON55L/M**				
L/L	6(38%)	28(27%)	0.62	
L/M	8(50%)	62(60%)		
M/M	2 (12%)	14 (13%)		
*Allele frequency*				
L	20(63%)	118(57%)	0.27	1.27 (0.56–3.01)
M	12(37%)	90(43%)		

**PON192Q/R**				
Q/Q	2(12%)	14(13%)	**0.0016 (0.005)**	
Q/R	8(50%)	84(81%)		
R/R	6(38%)	6(6%)		
*Allele frequency*				
Q	12(37%)	112(54%)	**0.045** (0.09)	1.94(0.23–1.11)
R	20(63%)	96(46%)		

(Bonferroni correction; Pc)

**Table 4:** Results of multivariate logistic regression analyses.

**Variables**	**β**	**p**	**OR**	**95% CI for OR**
LL	1,755	<0,001	5,783	2,579–12,967
Gender (Male)	1,548	<0,001	4,703	2,096–10,533
HT	2,179	<0,001	8,839	3,911–19,975
DM	0,938	0,069	2,554	0,931–7,003
HDL-C	−0,027	0,102	1,027	0,995–1,061
Fibrinogen	0,021	<0,001	1,021	1,013–1,030

## Discussion

RVO has been associated with various systemic vascular disorders, such as arteriosclerosis, diabetes mellitus, and dyslipidemia [15]. The mechanical compression of the veins at the arteriovenous crossings predisposes retinal veins to thrombus formation due to sclerotic changes in the retinal arteries. The increased rigidity of the arterial wall associated with these diseases may result in compression of retinal veins [16]. In addition, a case-control study reported decreased serum levels of paraoxonase-1 arylesterase activity, increased hyperhomocysteinemia, and increased oxidative stress in young adult patients with CRVO [4]. Many studies have also shown that RVO is a multifactorial disease and that genetic and environmental factors may contribute to its pathogenesis [17,18]. The present study evaluated the association between *PON1* L55M and Q192R polymorphisms and risk of RVO. While we found an association between the *PON1* L55M polymorphism and RVO, no association was identified between *PON1* Q192R and RVO. The frequency of the *PON1* 55 LL genotype was significantly lower in patients with RVO than in the control subjects (28% versus 55%; p=0.005).

Many studies have found associations between *PON1* polymorphisms and diabetic retinopathy. In a previous study, the allele frequency of leucine 54(L) was significantly higher in retinopathy patients than in those without retinopathy. However, *PON1* Gln192Arg frequency was not associated with diabetic retinopathy in insulin-dependent diabetes mellitus [9]. In addition, another study found that the LL genotype was closely associated with the presence of retinopathy. These data show that young people with type 1 diabetes and the LL polymorphism at position 54 of the *PON1* gene are more susceptible to retinal complications [19]. In contrast, no association between complications and *PON1* polymorphisms was found among Japanese patients with type 2 diabetes, but the results showed that *PON1* activity was higher for the RR and LL genotypes than for the other genotypes of each polymorphism [20]. Additionally, no association between *PON1* or *PON2* polymorphisms and retinopathy was found in a group from Manchester, England [21]. The variants of *PON1* have been studied in age-related macular degeneration (AMD) with controversial results. In a previous study, a positive association between the paraoxonase 192 RR and the paraoxonase 54 LL genotypes and wet AMD was demonstrated in Japanese patients [22]. In additional studies, the L55M and Q192R single nucleotide polymorphisms of the *PON1* gene were not associated with end stage AMD in Caucasian patients from Northern Ireland and Melbourne, Australia, with UK ancestry [23]. These data suggest that *PON1* genotypes vary among different ethnic groups and diseases. In another study, a weak association between *PON1* L55M and increased risk of wet AMD was reported [24]. Furthermore, Esfandiary et al. reported that *PON1* genotype frequencies were not significantly different among patients with AMD and controls [25]. In the present study, we showed that the *PON1* L55M polymorphism is significantly associated with RVO, while the *PON1* Q192R polymorphism is not. Previous studies [26-28]suggest an association between the *PON1* polymorphisms and coronary heart disease (CHD). The *PON1* 192 QQ-HDL polymorphism was most efficient at protecting LDL against oxidative modification, and the *PON1* RR-HDL polymorphism was least efficient. Individuals homozygous for the Q and M polymorphisms may be least susceptible to CHD, as these polymorphisms are most efficient at protecting LDL, and individuals homozygous for the R and L polymorphisms may be more susceptible and have the least protection [29]. *PON1* Q and *PON1* R may exhibit different affinities for lipid peroxides. A study has demonstrated that *PON1* Q has a greater capacity to protect against copper-ion induced LDL oxidation than *PON1* R 192 [30].

To the best of our knowledge, this is the first report on the association between *PON1* polymorphisms and RVO. Using a regression model, we determined that hypertension, male gender, higher plasma fibrinogen levels, and the lower frequency *PON1* 55 LL genotypes were positive risk factors for RVO. Most studies described a positive association between these factors and RVO. In a meta-analysis of 21 studies, O’Mahoney et al. reported that prevalence of systemic hypertension was 63.6% in patients with RVO compared with 32.6% in controls [31]. Diabetes mellitus was slightly more prevalent among cases with RVO (14.6%) than controls (11.1%). Fibrinogen levels were significantly higher in patients with RVO compared with the controls [32-34]. However, Gumus et al. reported that there was no statistically signiﬁcant difference regarding ﬁbrinogen in RVO [35].

Environment factors, such as smoking [36], a high-fat diet [37], and genetic polymorphisms, may affect *PON1* expression and activity. However, *PON1* genetic polymorphisms can be affected by environmental factors and ethnic variabilities. In addition, subjects with the LL genotype may have had higher *PON1* activity in our study groups, and thus, patients with RVO, who had a lower frequency of the LL genotype, may be more susceptible to LDL oxidation and atherosclerosis in the retinal arteries. Therefore, increased rigidity of the artery walls at the arteriovenous crossings may predispose retinal veins to thrombus formation and, thus, to RVO.

In our study, we were limited by an inability to measure paraoxonase concentrations and enzyme activity to test the effects of different genotypes in patients. Further studies of *PON1* activity associated with *PON1* polymorphisms are needed.

In summary, our data support that the *PON1* L55M gene polymorphism, but not the *PON1* Q192R polymorphism, is associated with RVO in a Turkish population. Given the complex nature of genetic susceptibility to retinal vascular occlusive diseases, further studies should be conducted on individual ethnic groups to verify the relationship between the 55L variant and disease.
